# Microbial divergence and evolution. The case of anammox bacteria

**DOI:** 10.3389/fmicb.2024.1355780

**Published:** 2024-02-13

**Authors:** Alba Cuecas, M. Julia Barrau, Juan M. Gonzalez

**Affiliations:** Institute of Natural Resources and Agrobiology, Spanish National Council for Research, IRNAS-CSIC, Sevilla, Spain

**Keywords:** anammox, hydrazine dehydrogenase, phylogeny, microbial evolution, genome divergence, diversification

## Abstract

Species differentiation and the appearance of novel diversity on Earth is a major issue to understand the past and future of microbial evolution. Herein, we propose the analysis of a singular evolutive example, the case of microorganisms carrying out the process of anammox (anaerobic ammonium oxidation). Anammox represents a singular physiology active on Earth from ancient times and, at present, this group is still represented by a relatively limited number of species carrying out a specific metabolism within the Phylum Planctomycetota. The key enzyme on the anammox pathway is hydrazine dehydrogenase (HDH) which has been used as a model in this study. HDH and rRNA (16S subunit) phylogenies are in agreement suggesting a monophyletic origin. The diversity of this singular phylogenetic group is represented by a few enriched bacterial consortia awaiting to be cultured as monospecific taxa. The apparent evolution of the HDH genes in these anammox bacteria is highly related to the diversification of the anammox clades and their genomes as pointed by phylogenomics, their GC content and codon usage profile. This study represents a clear case where bacterial evolution presents a paralleled genome, gene and species diversification through time from a common ancestor; a scenario that most times is masked by a web-like phylogeny and the huge complexity within the prokaryotes. Besides, this contribution suggests that microbial evolution of the anammox bacteria has followed an ordered, vertical diversification through Earth history and will present a potentially similar speciation fate in the future.

## 1 Introduction

Anaerobic ammonium oxidation (anammox) (Broda, 1977) is carried out by a specific group of bacteria with great significance in the global biogeochemical N-cycle (Kuypers et al., 2018). Anammox bacteria accounts for a 30–70% of all N_2_ released into the atmosphere (Lam and Kuypers, 2011; Oshiki et al., 2016; Stein and Klotz, 2016). Thus, anammox bacteria have been widely used in wastewater treatment plants for the removal of fixed nitrogen loads (Kartal et al., 2010; Stein and Klotz, 2016; Li et al., 2020) which indicates a major relevance in today’s world economy and sustainability. So far, the known anammox bacteria belong to the Phylum Planctomycetota (Wang et al., 2019) which includes seven proposed candidate taxa based on 16S rRNA gene sequences: “Candidatus Brocadia,” “*Ca.* Kuenenia,” “*Ca.* Jettenia,” “*Ca.* Scalindua,” “*Ca.* Anammoximicrobium,” “*Ca.* Anammoxoglobus” and “*Ca.* Bathyanammoxibiaceae” (Zhang and Okabe, 2020; Liao et al., 2022; Zhao et al., 2022). Because the culturing as monoespecific culture of these bacteria is arduous, today, most information on these bacteria has been obtained through whole-genome sequencing (WGS), including MAGs (Metagenome Assembled Genomes), of bacterial consortia and assemblages from nature or bioreactors (Liao et al., 2022).

The anammox process involves the use of nitrite or nitric oxide and ammonium to end by releasing N_2_ to the atmosphere. The enzyme performing at the final step of the anammox pathway is hydrazine dehydrogenase (HDH) which represents the key enzyme of the process and it is present in all anammox-performing bacteria (Maalcke et al., 2016; Akram et al., 2019; Liao et al., 2022). Hydrazine is a key intermediary (Kartal et al., 2011) formed, today, mainly by a biotic process (Dietl et al., 2015; Maalcke et al., 2016) or, in the ancient Earth, potentially by abiotic reactions (Folsome et al., 1981; Jia et al., 2021).

In spite of their relevance, scarce information is available on the evolutionary history of anammox bacteria. A recent study by Liao et al. (2022) suggests the origin of anammox bacteria on Earth around the Great Oxygenation Event, about 2.5 billion years ago. This places anammox bacteria close to the diversification of nitric reductase into the use of NO and O_2_ as major substrates and the origin of aerobic respiration (Ducluzeau et al., 2008; Santana et al., 2017). The transition from anaerobic respiration and the dominance of anaerobic processes (including denitrification) to an increasing significance of aerobic processes (including aerobic metabolisms) represented a major milestone in the history of the biogeochemical N-cycle on Earth. Specifically, as a consequence of increasing O_2_ concentration, the diversification of ammonium oxidizing processes could appeared around that time, gaining importance the aerobic ammonium oxidizing microorganisms (including ammonium-oxidizing bacteria, ammonium-oxidizing archaea and commamox species carrying out the nitrification process) and likely limiting the expansion of the anammox bacteria restricted to anoxic niches including the oxygen-free side of anoxic-oxic boundaries. At their initials, anammox appeared to be related to anoxic, oxygen-poor, niches and environments (Santana et al., 2017; Liao et al., 2022). Thus, the anammox bacteria constitute an ancient bacterial group maintaining their singularity through time (Wang et al., 2019; Liao et al., 2022) and preserving their activity in spite of the numerous changes undergone on Earth. At present, anammox bacteria remain as a unified metabolic and phylogenetic bacterial group which are unique in their capability of carrying out the anammox process and directly releasing to the atmosphere around 30–70% of fixed nitrogen forms as N_2_ (Lam and Kuypers, 2011; Oshiki et al., 2016; Stein and Klotz, 2016).

At present, microbial phylogeny and evolution present high complexity due to the huge microbial diversity existing on Earth (Curtis et al., 2002). In addition, numerous processes are leading to horizontal gene transfer (HGT) in the prokaryotes, such as mobile genetic elements, viruses, transposition events, among others (Johnston et al., 2014; Cuecas et al., 2017; Abe et al., 2020). Consequently, microbial phylogeny is often represented by web-like trees (Doolittle, 2000; Hug et al., 2016) showing an increased level of complexity to account for the frequent HGT events occurring through evolution among the prokaryotes (Goldman and Kaçar, 2022). Anammox can be selected as a singular group maintaining its uniqueness through evolution from ancient Earth. Thus, the anammox bacteria, and specifically their key HDH enzyme-encoding genes, can be an excellent case study for the analysis of gene, genome and species divergence through evolutionary history with perspectives to future diversification. The aim of this study is to analyze the divergence among the anammox bacteria as a unique metabolic and phylogenetic bacterial group that remains relatively independent of the rest of prokaryotes, as suggested by the related evolution of the HDH genes and the anammox bacterial genomes.

## 2 Data sources and methods

A blastp search was carried out at the NCBI^1^ against the non-redundant protein database (nr) using as reference the protein sequence of the enzyme hydrazine dehydrogenase from *Ca.* Kuenenia stuttgartiensis CSTR1 (Accession number QII14076.1). HDH gene sequences satisfying more than 85% coverage, 70% identity and an available genome sequence (either from WGS or MAG, >2 Mbp) were selected for further analysis. The Phylogenomic analysis was performed using get_HOMOLOGUES with the bidirectional best-hit algorithm (Contreras-Moreira and Vinuesa, 2013). Sequences were aligned using ClustalW (Thompson et al., 1994). Phylogenies were generated with MEGA X (Kumar et al., 2018) using the Neighbor-Joining method and the Poisson model. Bootstrap values are expressed as percentages corresponding to 1,000 trials. GC content and frequency of codon usage were calculated using own software. Non-metric multidimensional scaling (NMDS) using Bray distances was performed using PAST (Hammer and Harper, 2001) on the frequency of codon usage of genes and genomes.

## 3 Phylogeny

From the discovery of the anammox bacteria in the early 1990s, a major preliminary taxonomic classification of Candidatus (*Ca.*) taxa and the clades within the anammox bacteria have been envisioned from the analysis of 16S rRNA gene sequencing (Kuenen, 2008; Liao et al., 2022). Due to the lack of monospecific cultures in this singular bacterial group, those 16S rRNA gene sequences have been retrieved from bacterial enrichments, consortia and assemblages either from natural environments or the bioreactor assays pursuing an increased of the anammox process with views aimed to applications mostly related to waste treatments. Most additional information on anammox bacteria have been based on whole genome sequencing (WGS) or metagenomic assembled genomes (MAGs) from those sources of anammox bacteria (Kuenen, 2008; Liao et al., 2022).

Based on 16S rRNA gene sequence analysis, the anammox bacteria have been classified within the Phylum Planctomycetota and into a total of seven major groups, candidate to future taxa: “Candidatus Brocadia,” “*Ca.* Kuenenia,” “*Ca.* Jettenia,” “*Ca.* Scalindua,” “*Ca.* Anammoximicrobium,” “*Ca.* Anammoxoglobus” and “*Ca.* Bathyanammoxibiaceae” (Zhang and Okabe, 2020; Lodha et al., 2021; Liao et al., 2022; Zhao et al., 2022). This uniqueness of anammox bacteria among the broad prokaryotic diversity suggests certain independence and evolutive isolation between the anammox bacteria and all other prokaryotes, at least, in relationship to their genome history. Besides, this uniqueness represents an evidence to propose a monophyletic view for this bacterial group, also in agreement to previous reports (Kuenen, 2008; Liao et al., 2022).

**Figure 1:**
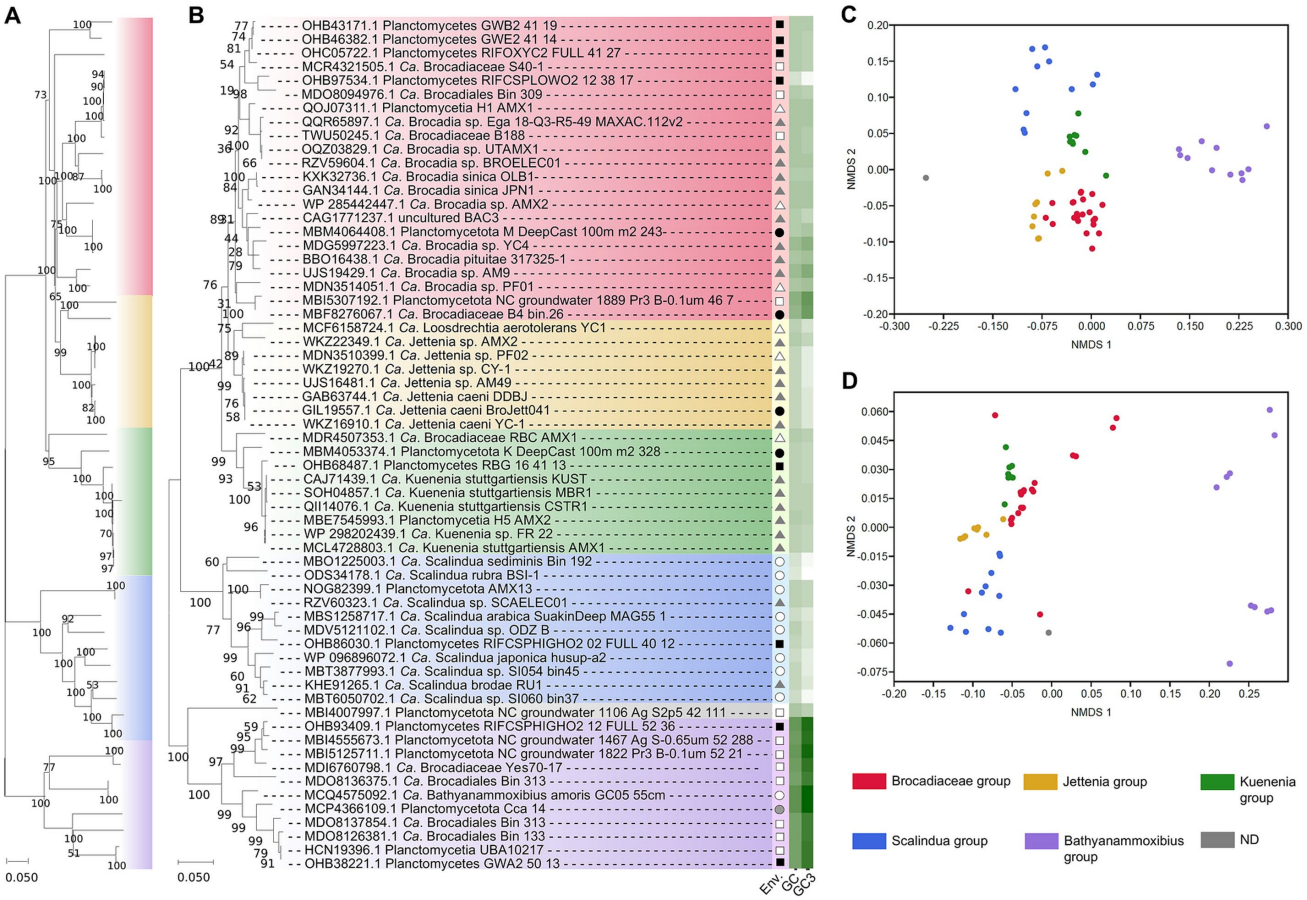
Diversification of anammox bacteria based on phylogenomics (WGS and MAGs) **(A)** and based on HDH-encoding gene sequences **(B)**. The origin of these sequences is indicated in the column “Env.” by the following symbols: soil (black square), ground water (open square), freshwater (black circle), marine and saline environment (open circle), bioreactors (black triangle), wastewater (open triangle), and animal isolate (gray-filled circle). The GC content (GC) and the GC content at the third codon position (GC3) is indicated in a white (lower values) to green (higher values) color scale. NMDS analyzes show a distinctive distribution of clades and they are based on the frequency of codon usage in HDH-encoding genes **(C)** and anammox bacterial genomes **(D)**. A total of 52 genome assemblies including 21 orthologs have been used to construct the phylogenomic dendrogram **(A)**. A total of 62 HDH-encoding gene sequences constitute the HDH-based dendrogram **(B)** with an approximate sequence length of 700 bp. The color code [used in **(A–D)**] to differentiate the major clades distinguished in the analyzes is shown at the bottom-right. Bootstrap values represent percentages from 1,000 trails.

Assuming a highly likely monophyletic origin of anammox, the major genes involved in the anammox pathway should show as well a similar evolutive pattern. We are not aware of a phylogeny for the key enzyme of the anammox pathway, HDH (Liao et al., 2022) and our results (Figure 1) suggest a paralleled phylogeny of 16S rRNA gene dendrogram (Kuenen, 2008; Lodha et al., 2021; Liao et al., 2022) and that generated for the HDH gene sequences. This evidence supports a similar evolutive history of the anammox bacteria and their HDH genes. Previously, Harhangi et al. (2012) showed a similar parallelism between the 16S rRNA gene phylogeny and the hydrazine synthase-encoding genes in these bacteria which further support the hypothesis of a similar evolutive history for these bacteria and their major anammox enzyme-encoding genes. Thus, results suggest certain evolutive isolation from other bacterial phyla and even from non-anammox Planctomycetota bacteria (Kuenen, 2008; Liao et al., 2022).

At present, the anammox bacteria shows a fairly independent phylogeny with scarce HGT influence (so far unreported) from other bacteria, unlike most other bacterial phyla (Cuecas et al., 2017; Sheinman et al., 2021; Sengupta and Azad, 2022). This singularity within the complexity of bacterial phylogeny at present time represents an additional piece of evidence in support of a monophyletic origin of the anammox-performing bacteria previously shown on the basis of 16S rRNA (Kuenen, 2008; Lodha et al., 2021; Liao et al., 2022) and in this study through the HDH-encoding gene sequences and phylogenomics (Figure 1).

A monophyletic origin for the anammox suggests the existence, at some time point, of a last common ancestor (LCA) for the anammox bacteria. The LCA for the anammox bacteria has been proposed to be dated back to around 2.5 billion years (Liao et al., 2022). This ancient date suggests a major relevance of anammox throughout our planet history and their potential contribution to the biogeochemical processes on Earth. In addition, current biotechnological interests, specifically related to the transformation of fixed nitrogen forms during waste treatments highlights the importance of these bacteria for a sustainable economy.

## 4 Genes and genomes divergence

Analyzes of GC content and codon usage in the HDH-encoding genes and their genomes (mostly MAGs) (Figure 1) suggests equivalent evolutive patterns of divergence differentiating similar clades. Non-metric multidimensional scaling analyses show that codon usage in the HDH-encoding genes and their genome sequences present related divergence because similar grouping is obtained. Codon usage of the HDH-encoding genes and the available anammox genome annotated sequences suggest that a paralleled evolutive divergence is taking place leading to differentiating clades (mainly corresponding to the proposed candidate genera) within the anammox bacteria. The results (Figure 1) suggest that the anammox bacteria are slowly and progressively diverging which can be observed from the different clades detected in this singular group. Similar diversification can be observed when analyzing, using a similar procedure, the HDH-encoding genes. These observations suggest that a key anammox gene and the anammox bacterial genomes show a similar evolutive divergence along Earth history. This parallelism might imply that similar or random evolutive mechanisms influence the differentiation of the genomes of these candidate bacterial taxa and their key enzyme (HDH)-encoding gene. If this is the case, which needs to be confirmed through further analyzes, the evolutive fate of the anammox bacteria and their key enzyme (HDH) might diverge progressively following specific whole-genome wide trends (to be determined) likely governed by singular environmental and specific niche requirements.

Among the anammox bacteria, different niche preferences have been reported for the different anammox clades within the group (Lam and Kuypers, 2011; Zhang and Okabe, 2020; Zhao et al., 2023) (Figure 1). The ecological distribution of anammox bacteria presents a strong dependence on the environment (Oshiki et al., 2016; Zhang and Okabe, 2020). Anammox are commonly found at the oxic-anoxic interface in a variety of environments (Jensen et al., 2008; Seuntjens et al., 2018; Liao et al., 2022). Salinity is a factor affecting anammox distribution. For example, “*Ca.* Scalindua” dominate saline environments (Sonthiphand et al., 2014) and “*Ca.* Brocadia” and “*Ca.* Kuenenia” have been mostly located to non-salty environments such as soils and freshwater systems (Zhang and Okabe, 2020). The anammox bacteria also show niche differentiation among clades. Temporal and spatial differential distribution have been reported for “*Ca.* Brocadia” and “*Ca.* Kuenenia.” The characteristic dependency of anammox bacteria on fixed nitrogen sources (nitrite and ammonium) determines species distribution (van der Star et al., 2007; Pitcher et al., 2011) as well as the availability of additional C sources (Zhao et al., 2022, 2023).

Although the anammox bacteria show a number of clades when attempting phylogenetic analyzes either on their genes or genomes, assuming the long evolutive time (*ca.* 2.5 billion years) required to show these differences suggests a fairly slow progression towards the divergence of the anammox clades adapting to specific niches. The uniqueness of the anammox process and the relative independence of this phylogenetic group from other bacteria could suggest than the evolutive fate of the anammox might be fairly conserved and maintained with a minor and slow changing rate over time; mainly because of a specific adaptation to a singular type of niches. Nevertheless, at present, there is no additional information to predict the potential evolutive fate of the anammox bacteria.

## 5 Discussion

The anammox bacteria represent a singular bacterial group showing relative isolation within the prokaryotes. So far, their evolutive history suggests that this group potentially evolves as a result of environmental constrains presenting minimum influences from other bacterial phyla such as through HGT-type for accelerated changes. As well, to our knowledge, no phages have been reported for the anammox bacteria perhaps a consequence of their low growth rate. These points suggest the existence of genomes showing less genomic plasticity than shown for most other bacterial phyla (Johnston et al., 2014; Cuecas et al., 2017; Sheinman et al., 2021) although some genome variability within the anammox bacteria has been reported (Ding and Adrian, 2020). While this is a hypothesis that remains to be tested, present information points to this putative scenario suggesting a study case for genomic analyzes and the evolutive history of this bacterial group.

It is expected that future studies and additional microbial diversity surveys will greatly enhance the today’s relatively limited diversity within the anammox bacteria. Currently, a limiting sampling on anammox bacteria precludes to realize the actual diversity for the anammox bacteria existing on Earth (Harhangi et al., 2012; Maalcke et al., 2016; Liao et al., 2022) in a similar way that the whole prokaryotic diversity on Earth remains to be truly understood (Curtis et al., 2002). Potential issues with the slow growth rates of the anammox bacteria, potential discrimination during PCR amplification on microbial diversity surveys, added to the difficulty for culturing and the lack of monospecific cultures all sum up to make this singular bacterial group a difficult target for further research.

Diversification and speciation among prokaryotes are major research lines to be developed in the years to come. It is expected that major advances on understanding these processes will be gained during the next decade or so. Herein, we propose that the anammox bacteria could represent a singular group, both on their metabolism and evolutive history, to analyze specific phenomena related to those issues within the prokaryotes. In the anammox bacteria, different aspects converge to point this group as a singular target for evolutive studies, most importantly, a time-located LCA, their slow evolution, a common genome-wide divergence trend and the apparently scarce influence on gene exchange from other bacterial groups.

As pointed above, the environment, and specific niche requirements, might represent major evolutive factors influencing the divergence of the identified anammox clades. Currently, different niches has been reported for some of the different anammox clades which would confirm that the environment is having major influence on microbial evolution and specifically on the diversification of the anammox bacteria.

Due to the high relevance of the anammox bacteria in the environment, including their application in waste treatment plants, great interest exist on understanding the physiology, ecology, evolution and behavior of this singular bacterial group. This perspective contributes to propose the anammox bacteria as a unique case study for the analysis of evolutive trends among bacteria and specifically within the anammox-performing bacteria. Current information, mostly from WGS and MAGs, suggests slow and independent evolution which appears to be mostly forced by environmental constrains. Future research on the development of these hypotheses will confirm the role and fate of anammox bacteria, and, from those results, the extracted knowledge con assist to comprehend the evolutive history of other bacterial phyla (Goldman and Kaçar, 2022).

## Data availability statement

Publicly available datasets were analyzed in this study. This data can be found at: NCBI, https://blast.ncbi.nlm.nih.gov/.

## Author contributions

AC: Conceptualization, Formal analysis, Methodology, Writing – review & editing. MB: Formal analysis, Methodology, Writing – review & editing, Data curation. JG: Formal analysis, Methodology, Conceptualization, Funding acquisition, Supervision, Writing – original draft.

## Footnotes

^1^ https://blast.ncbi.nlm.nih.gov/Blast.cgi
